# Methylglyoxal-Scavenging Enzyme Activities Trigger Erythroascorbate Peroxidase and Cytochrome c Peroxidase in Glutathione-Depleted *Candida albicans*

**DOI:** 10.4014/jmb.2010.10057

**Published:** 2020-11-16

**Authors:** Sa-Ouk Kang, Min-Kyu Kwak

**Affiliations:** 1 Laboratory of Biophysics, School of Biological Sciences, and Institute of Microbiology, Seoul National University, Seoul 08826, Republic of Korea; 2 Department of Food and Nutrition, Institute of Food and Nutrition Science, Eulji University, Seongnam 13135, Republic of Korea

**Keywords:** Alcohol dehydrogenase 1, *Candida albicans*, erythroascorbate peroxidase, glutathione, methylglyoxal, NAD(H)-linked methylglyoxal oxidoreductase

## Abstract

γ-Glutamylcysteine synthetase (Gcs1) and glutathione reductase (Glr1) activity maintains minimal levels of cellular methylglyoxal in *Candida albicans*. In glutathione-depleted *Δgcs1*, we previously saw that NAD(H)-linked methylglyoxal oxidoreductase (Mgd1) and alcohol dehydrogenase (Adh1) are the most active methylglyoxal scavengers. With methylglyoxal accumulation, disruptants lacking *MGD1* or *ADH1* exhibit a poor redox state. However, there is little convincing evidence for a reciprocal relationship between methylglyoxal scavenger genes-disrupted mutants and changes in glutathione-(in)dependent redox regulation. Herein, we attempt to demonstrate a functional role for methylglyoxal scavengers, modeled on a triple disruptant (*Δmgd1*/*Δadh1*/*Δgcs1*), to link between antioxidative enzyme activities and their metabolites in glutathione-depleted conditions. Despite seeing elevated methylglyoxal in all of the disruptants, the result saw a decrease in pyruvate content in *Δmgd1*/*Δadh1*/*Δgcs1* which was not observed in double gene-disrupted strains such as *Δmgd1*/*Δgcs1* and *Δadh1*/*Δgcs1*. Interestingly, *Δmgd1*/*Δadh1*/*Δgcs1* exhibited a significantly decrease in H_2_O_2_ and superoxide which was also unobserved in *Δmgd1*/*Δgcs1* and *Δadh1*/*Δgcs1*. The activities of the antioxidative enzymes erythroascorbate peroxidase and cytochrome c peroxidase were noticeably higher in *Δmgd1*/*Δadh1*/*Δgcs1* than in the other disruptants. Meanwhile, Glr1 activity severely diminished in *Δmgd1*/*Δadh1*/*Δgcs1*. Monitoring complementary gene transcripts between double gene-disrupted *Δmgd1*/*Δgcs1* and *Δadh1*/*Δgcs1* supported the concept of an unbalanced redox state independent of the Glr1 activity for *Δmgd1*/*Δadh1*/*Δgcs1*. Our data demonstrate the reciprocal use of Eapx1 and Ccp1 in the absence of both methylglyoxal scavengers; that being pivotal for viability in non-filamentous budding yeast.

## Introduction

Methylglyoxal (MG, CH_3_COCHO) reacts with nucleic acids and proteins, causing to cellular damage [1], and also inhibits cell division in eukaryotes [2]. MG non-specifically binds to amines, amino acids, and proteins and produces biologically active free radicals [3-5]. Advanced glycation end-products are derived from MG, leading to hyperglycemic damage in cells [1, 6]. This highly reactive α-ketoaldehyde inhibits human leukemia 60 cells, resulting in apoptosis [7]. In the fission yeast *Schizosaccharomyces pombe*, MG can activate stress-activated protein kinase signaling cascade [8].

As demonstrated in our previous studies at the microbial level, MG accumulation in cells results in the defective growth and the arrest of the G1-phase specific cell cycle in γ-glutamyl cysteinyl synthetase (*GCS*)-deficient *gcs*^-^ of *Dictyostelium discoideum* [9]. Glutathione (GSH)-depleted strains provide a working concept for cellular accumulation of MG via the inactive GSH-required glyoxalase system [9]. Investigations have focused on a number of MG scavengers, including aldehyde dehydrogenase, aldehyde reductase, aldose reductase, and α-ketoaldehyde dehydrogenase. These enzymes commonly catalyze the oxidation or reduction of MG into pyruvate or acetol [10]. Inspired by these findings, in *Candida albicans*, we previously purified and characterized two predominant MG-scavenging enzymes, NAD(H)-linked alcohol dehydrogenase 1 (CaO19.3997; Adh1) [11] and NAD(H)-linked MG oxidoreductase (CaO19.4309; Mgd1) [12]. The Mgd protein, Mgd1, has been revealed to be CaO19.4309 of *C. albicans* SC5314 Assembly 22 [12]. This oxidoreductase was a glycine-rich protein (Grp2) associated with the Gre2 family likely as a putative NAD(P)H-dependent MG reductase (http://www.candidagenome.org/). The revealed common mechanism of activities of these enzymes is that Mgd1 and Adh1 activities catalyze the oxidation and reduction of MG to pyruvate and acetol, respectively. Disruption of the genes encoding NAD(H)-linked Mgd1 and Adh1 leads to defects in cell growth, differentiation, and G1-phase cell-cycle arrest, accompanied by accumulations of MG and pyruvate with mitochondrial inactivity [11]. *ADH1* disruptants, constructed of both the wild-type and GSH-depleted *Δgcs1* (*C. albicans* *GCS1*), display supraphysiological increase in MG, as well as severe defects in growth, differentiation, and full virulence. Interestingly, Mgd1 activity scavenges both MG and pyruvate to maintain metabolic homeostasis and redox buffers via the triggering of a combination of glycolytic enzymes and Adh1 expression.

However, there is little data concerning cellular MG content-dependent oxidative stress-responsive enzyme activity alteration in *C. albicans* [12]. Reactive oxygen species (ROS) are formed from the reduction of one or two electrons of molecular oxygen creating hydroxyl radicals (^•^OH), superoxide anions (O_2_^–^), and hydrogen peroxide (H_2_O_2_) [13]; all being causative factors in DNA mutations and genomic instability [14]. Although convincing evidence for MG-triggered ROS production and/or ROS-mediated MG content control is lacking, the scavenging mechanisms of ROS have been sufficiently elucidated; the superoxide anions scavenged by superoxide dismutases (Sods) generate the simplest form of peroxide, H_2_O_2_, which is detoxified by catalase (Cat) and/or GSH reductase (GR) [15, 16]. ROS, antioxidative enzyme activities, and their resulting metabolite products have been postulated. Studies on redox regulatory mechanisms have linked them to cell growth, proliferation, division, and differentiation in eukaryotic microorganisms [13] including *C. albicans* [11, 12, 17, 18]. Partial experimental data concerning cellular MG-derived ROS generation, in the absence of the antioxidative activities of several enzymes, comes from previously observed MG and ROS accumulations, with resulting defects in growth, filamentation, and virulence [12, 19, 20]. These include erythroascorbate peroxidase (CaO19.584, Eapx1) [19], cytochrome c peroxidase (CaO19.7868, Ccp1) [20], Gcs1 (CaO19.5059) [21], and Glr1 (CaO19.4147) [18]. However, at present there is little data regarding the effects of the absence of the MG scavengers Mgd1 and Adh1 in GSH-depleted states on the production of ROS, or the resulting physiology during proliferation. In this study, using experiments based on GSH-depleted non-hyphae-inducing cells independent of GSH metabolism, we examine the cellular MG-derived enhanced antioxidative enzyme activities resulting from a lack of representative MG scavengers using the triple disruptant *Δmgd1*/*Δadh1*/*Δgcs1*. Thus, using measurements of redox metabolites and MG, we demonstrate how antioxidative actions by *C. albicans* enzymes contribute to redox regulation.

## Materials and Methods

### Strains and Growth Conditions

The *C. albicans* strains used in this study are listed in Table 1. To culture *C. albicans* cells harboring disrupted genes, Ura^+^ and Ura^–^ strains were cultured in minimally defined SD and YPD media [21], respectively. YPD medium (1% yeast extract, 2% peptone, 2% glucose) or minimally defined SD (2% glucose, 0.5% ammonium sulfate, 0.17% yeast nitrogen base without amino acids and ammonium sulfate) medium and appropriate supplements in liquid broth or 1.8% agar-containing plates were prepared and utilized by means described previously [22]. Unless otherwise stated, all physiological and biochemical experiments employed Ura^+^ heterozygous strains. All Ura^+^-strains were commonly grown in SD broth and inoculated at an optical density of 1×10^6^ cells/ml at 28°C [22]. GSH depletion from the Ura^+^ *GCS1* disruptant and its derivatives was performed using previously detailed methods [9, 21].

### *ADH1* and *MGD1* Disruption

*MGD1* and *ADH1* disruption was conducted in *Δgcs1* using known means [11, 12]. An *MGD1* and *ADH1* triple disruptant derived from the MK604 (*Δmgd1*/*Δgcs1*) strain was constructed to create MK803 (*Δmgd1*/*Δadh1*/*Δgcs1*). Both genes were commonly disrupted using the URA blaster method employing pQF181 and its derivative pQF182 to remove the *MGD1*- and *ADH1*-coding regions, respectively [23-25]. The primers and plasmids used in this study are detailed in Table 1. Using YB204, a homozygous Ura- *Δgcs1*, spontaneous Ura^+^ strains from heterozygous MK601 ((*MGD1*/*mgd1*)/*Δgcs1*), MK701 ((*ADH1*/*adh1*)/*Δgcs1*), and MK801 (*Δmgd1*/(*ADH1*/*adh1*)/*Δgcs1*) disruptants were selected on SD plates supplemented with 5-fluoroorotic acid to reveal the URA3 locus using uracil prototrophy [25]. The resulting homozygous MK602 ((*MGD1*/*mgd1*)/*Δgcs1*), MK702 ((*ADH1*/*adh1*)/*Δgcs1*), and MK802 (*Δmgd1*/(*ADH1*/*adh1*)/*Δgcs1*) strains were confirmed to contain disrupted *ADH1* or *MGD1*, and to have lost the URA3 gene, through intrachromosomal recombination between tandem hph repeats. Using the same method described above, spontaneous recombination between the hph repeats involved selection on FOA plates and utilization for a second round of transformations. Ura^+^ transformants were then selected on uracil-deficient media, yielding MK603 (*Δmgd1*/*Δgcs1*), MK703 (*Δadh1*/*Δgcs1*), and MK803 (*Δmgd1*/*Δadh1*/*Δgcs1*). Unless otherwise stated, all the physiological and biochemical experiments were performed using Ura^+^ heterozygous strains (*i.e.*, YB203, *Δgcs1*; MK603, *Δmgd1*/*Δgcs1*; MK703, *Δadh1*/*Δgcs1*; MK803, *Δmgd1*/*Δadh1*/*Δgcs1*).

### Quantification of Quinoxaline Derivatives 

MG and pyruvate levels were measured by converting these metabolites into quinoxaline derivatives using 1,2-diaminobenzene (Merck) and methods already detailed [9, 26]. For utilization in enzymatic studies and intracellular quantification, MG from a commercial 40% solution was repurified by vacuum distillation (Sigma, USA) to eliminate polymerized MG and impurities [27]. The distilled MG concentration was calculated to be 0.5565 M, which was stoichiometrically confirmed by 2-methylquinoxaline (Sigma) using a high-performance liquid chromatography (HPLC) system. Analytical grade pyruvate (Sigma) was purchased and used without further purification. Quinoxaline derivatives of MG and pyruvate (*i.e.*, 2-methylquinoxaline and 2-hydroxy-3-methylquinoxaline) were obtained using a solid-phase extraction (SPE) cartridge (Waters) containing a C18 resin. The resulting derivatives were separated using an Agilent 1200 series HPLC system with a Zorbax Eclipse XDB-C18 analytic column (Agilent, 150 × 4.6 mm) at a wavelength of 336 nm with an internal standard 5-methylquinoxaline (Sigma).

### D-Erythroascorbic Acid and GSH Measurements

The intracellular D-erythroascorbic acid content was measured by employing known techniques [28, 29]. Samples were applied to an analytical HPLC system (Waters) that was equipped with a Waters 460 electrochemical detector. The resulting extracts were separated with ZORBAX SB-C18 columns (Agilent, 250 mm × 4.6 mm) and eluted with 0.1% trifluoroacetic acid at a flow rate of 0.7 ml/min.

Cellular GSH was labeled with monobromobimane (mBBr) and measured as described previously [30]. The mBBr-derivatized thiol compounds were analyzed by HPLC with a Hewlett-Packard 1050 series fluorescence detector with ZORBAX SB-C18 columns (Agilent, 250 mm × 4.6 mm). Samples were eluted with 0.1% trifluoroacetic acid at a flow rate of 0.7 ml/min. The mobile phase consisted of 15% methanol and 85% trifluoroacetic acid (0.1%) at a wavelength at 370 nm.

### Superoxide and H_2_O_2_ Measurements

*C. albicans* cells grown in liquid SD medium until the early (12 h) and late (24 h) exponential phases of cell growth were harvested, washed, and resuspended at 10^8^ cells/ml in 100 mM sodium phosphate buffer (pH 6). The intracellular superoxide content was measured using dihydroethidium with 18.9 μM dihydroethidine [31]. Superoxide anions were measured using a Cary Eclipse fluorescence spectrophotometer (Varian) with λex = 518 nm, λem = 605 nm.

Cellular peroxide was estimated using 19.5 μM 2′,7′-dichlorofluoroscein diacetate (H_2_DCFDA) with a Cary Eclipse fluorescence spectrophotometer (Varian) with λex = 495 nm and λem = 525 nm, with previously described methods [12].

### Enzyme Activity Assays

Cells were grown in minimally defined SD medium until the early (12 h) and late (24 h) exponential phases, harvested by centrifugation at 6,000 g for 15 min, and then homogenized using a Mini Bead-Beater (Biospec Products) for 90 s containing 1 mM phenylmethanesulfonyl fluoride. The protein concentrations of the *C. albicans* crude extracts were measured using known means [32]. Enzyme activity using crude extracts was commonly examined using continuous assays with a Hewlett-Packard (HP) 8452A diode array spectrometer (Palo Alto, USA). The cell lysates with 20 mg/mL crude protein were diluted 1/20 with buffers, and the enzyme reaction was observed using 10 μg crude protein lysates.

Glr1 activity was spectrophotometrically measured as previously described [33]. The enzyme reaction mixture consisted of 2 mM NADPH, 20 mM GSSH, and 10 μg crude extracts in 0.2 M potassium phosphate buffer (pH 7.4). The decrease in absorbance at 340 nm was monitored for 2 min. One unit (U) of Glr1 activity was defined as the micromoles of NADPH oxidized per minute per milligram of protein, with an NADPH ε = 6.2 mM^-1^×cm^-1^.

The Eapx1 activity was spectrophotometrically monitored based on L-ascorbic acid oxidation using H_2_O_2_ at 290 nm (ε = 2.8 mM^-1^ ×cm^-1^) [34]. The enzyme activity assay mixture consisted of 0.5 mM L-ascorbic acid and 0.1 mM H_2_O_2_, in 50 mM potassium phosphate (pH 7) [19].

Ccp1 activity was detected using previously detailed methods [35]. To avoid overlapping of enzyme activity with that of catalase owing to competition for H_2_O_2_, the samples were treated with the catalase activity inhibitor 20 mM aminotriazole prior to Ccp1 activity determination. Ccp1 activity was measured at 550 nm by observing the oxidation of dithionite-reduced cytochrome c. The enzyme reaction mixture consisted of 20 μM reduced cytochrome c in 50 mM sodium acetate buffer (pH 6). Cytochrome c reduction was observed at 550 nm, with an extinction coefficient (ε) of 27.7 mM^-1^ cm^-1^ mM−1. The enzyme activity reaction was performed by the addition of 0.1 mM H_2_O_2_. Ccp1 activity was tested at 550 nm by observing the oxidation of ferrocytochrome c (Δ_ε550_ = 19.6 mM^-1^ cm^-1^), as proposed previously [36]. One unit (U) is deﬁned as the amount of enzyme required to oxidize 1 μmol ferrocytochrome c/min.

Total peroxidase activity was spectrophotometrically measured by examining the oxidation of *o*-dianisidine dihydrochloride (Sigma-Aldrich) at 460 nm. The enzyme reaction mixture consisted of 0.3 mg *o*-dianisidine dihydrochloride/ml, 18 mM H_2_O_2_, and an appropriate amount of protein extract in 10 mM potassium phosphate buffer (pH 7). One unit (U) was equivalent to an increase of 11.3 units/min in A_460_, with an absorption coefficient of 11.3 M-1 cm^-1^ for *o*-dianisidine [37].

The enzyme activities of Cu–Zn Sod (Sod1) and Mn Sod (Sod2) were measured based on the inhibitory activities of xanthine oxidase-induced catalysis of xanthine to produce superoxide anion-free radicals. Superoxide radicals oxidize hydroxylamine to produce nitrite, which reacts with a developer to produce a purple color. The Cu–Zn Sod and Mn Sod activity assay kit (Nanjing Jiancheng Bioengineering Research Institute) was used to observe Sod1 and Sod2 activities, respectively. One unit (U) of Sod activity is commonly defined as the amount of Sod required to inhibit xanthine oxidation by 50% of proteins (U/mg).

The enzyme activity of MG oxidation and MG reduction by Mgd and MG reductase (Mgr) was measured in cell crude extracts based on the ability of the enzyme to catalyze the oxidation of MG in the presence of NAD^+^ and the reduction of MG in the presence of NADH [12]. The rate of MG oxidation and reduction was determined by an increase and decrease in absorbance at 336 nm using an NAD(H) ε = 6.22 mM^-1^×cm^-1^ and was expressed as the nanomoles (nmol) of NAD reduced and NADH oxidized (nmol·min^−1^·mg protein ^−1^). The MG-oxidizing reaction mixture commonly consisted of 2 mM MG and 1 mM NAD^+^ in 50 mM Tris-HCl buffer (pH 8.2) for MG oxidation; 2 mM MG and 1 mM NADH in 50 mM potassium phosphate buffer (pH 6.5) were used to observe MG reduction.

### Real-Time RT-PCR

Real-time PCR was performed using the SYBR Premix Ex TaqTM (TaKaRa) on Applied Biosystems 7300 Real-Time PCR systems. Total RNA extraction was performed using a beadbeated cell lysate with an RNAiso Plus (TaKaRa) Reagent; 5 μg of RNA was reverse-transcribed into cDNA using the SuperScript III Reverse Transcriptase Kit (Promega). The expression level of the target genes was normalized to that of *ACT1*. All primers used for the assay are indicated in Table 1.

### Statistical Analysis

Data are presented as the means ± standard deviation (Sd). The statistical significance of the differences was evaluated using the Student’s *t*-test and Microsoft Office Excel (2015). For all comparisons, *p < 0.05, **p < 0.01, and ***p < 0.001 were considered statistically significant.

## Results

### GSH-Deprived States in *Δmgd1*/*Δadh1*/*Δgcs1*

**Fig. 1:**
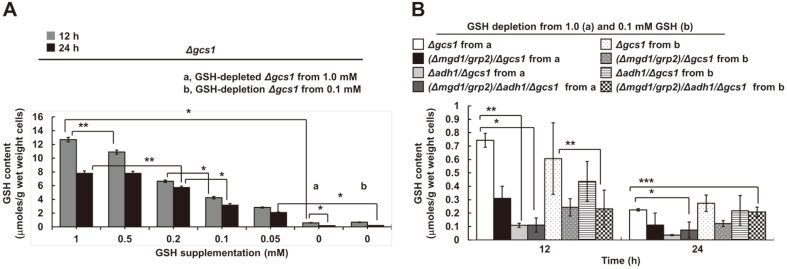
The effect of GSH deprivation on MK803 (*Δmgd1/Δadh1/Δgcs1*). (**A**) Cellular GSH uptake rates in SD liquid medium. The GSH content in this strain was measured by means described in the Materials and Methods section. (**B**) The cellular content of GSH in w *Δgcs1*-based disruptants. All Candida cells were grown on SD liquid broths for 24 h until the late exponential phase under the following conditions: a and b, 1 and 0.1 mM GSH-supplemented *Δgcs1*-based disruptants, transferred from a and b grown to the mid-exponential phase at 18 h, respectively. All experiments were conducted independently in triplicate. Data are represented as the mean ± standard deviation (Sd). Asterisks indicate statistically significant values (*p < 0.05, **p < 0.01, and ***p < 0.001).

Previously, near-normal cell growth of YB203 (*Δgcs1*) and *ΔgcsA*, by 1 mM GSH supplementation, was observed in *C. albicans* and *D. discoideum*, respectively [38, 39]. Based on this finding, to assess the effect of GSH depletion on cell viability of the triple disruptant MK803 (*Δmgd1*/*Δadh1*/*Δgcs1*) during cell proliferation, we tested the minimal amount of exogenous GSH required for yeast growth. This was observable via the rate of exogenous GSH uptake to YB203 (*Δgcs1*) (Fig. 1A). When considering intracellular GSH content in all disruptants by GSH uptake (Fig. 1B), it was seen that YB203 (*Δgcs1*) did not grow below 0.05 mM of GSH supplementation. Based on the GSH content of the wild-type cells (approximately 60.48 μmol per g wet weight cells) [12], GSH uptake levels required for the cell viability of MK803 (*Δmgd1*/*Δadh1*/*Δgcs1*) was revealed to be 0.05–0.1 mM of GSH supplementation in the case of the late exponential growth phase (24 h). These values corresponded to the amount of GSH per gram wet weight cells at 24 h. This result agreed with our previous findings of a critical threshold at the late exponential growth phase in the case of the wild-type and YB203 (*Δgcs1*) [12]. Experiments under the control of GSH supplementation revealed extremely low GSH uptake in YB203 (*Δgcs1*) (Fig. 1). This was very similar to results reported in prior studies conducted using *GCS* disruptant in other eukaryotic microorganisms, including *S. cerevisiae* *Δgsh1* [40] and *D. dictyostelium* *ΔgcsA* [41]. All of the GSH-depleted disruptants exhibited severe GSH-deficient states. Particularly, the GSH content in all disruptants transferred from 0.1 mM GSH-containing media were commonly near a below-critical threshold between 2.08 and 3.14 μmol. These GSH concentrations turned out to be under 0.05 and 0.1 mM GSH supplementation of GSH per gram wet weight cells at 24 h. Thus, further experiments were performed using all disruptants transferred from 1 and 0.1 mM GSH-supplemented media until the late-exponential phase at approximately 24 h.

### The Decreased NADH-Linked Mgr Activity in *Δgcs1*-Based Disruptants

**Fig. 2:**
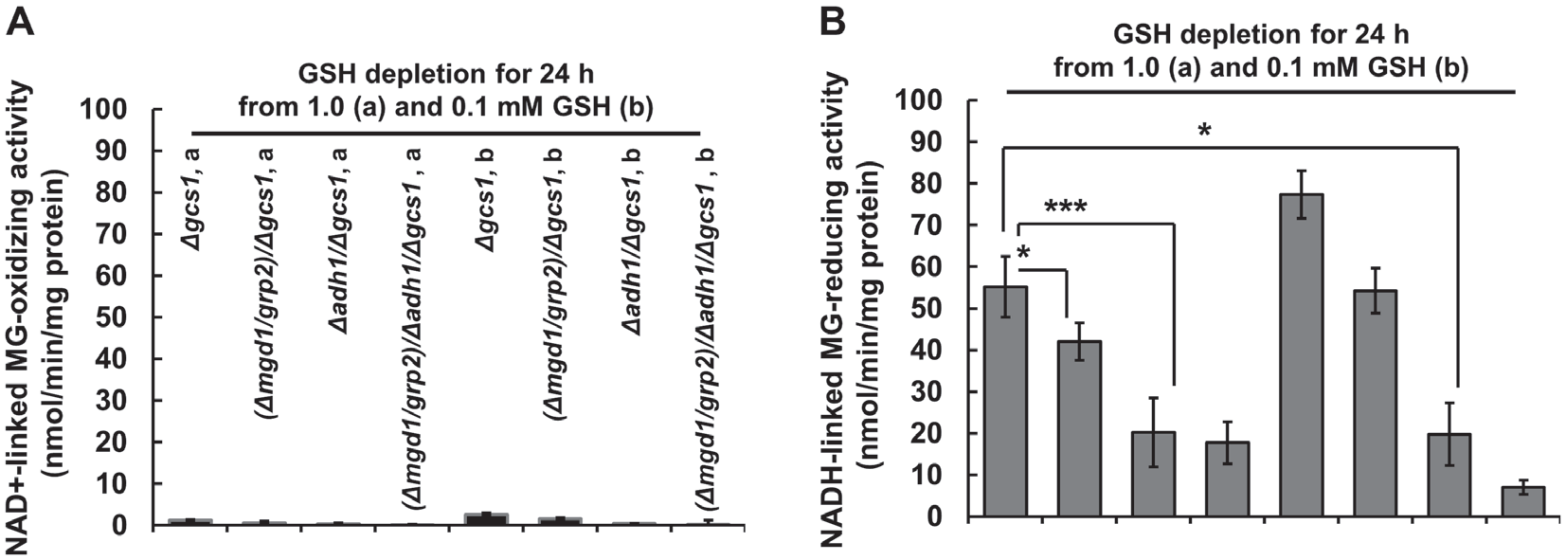
The effect of both *MGD1* and *ADH1* gene disruption on Mgd and Mgr activities in Δgcs1-derived MK803 (*Δmgd1/Δadh1/Δgcs1*). The activity of MG oxidation and reduction by (**A**) Mgd and (**B**) Mgr was observed based on the ability of the enzyme to catalyze the oxidation of MG in the presence of NAD^+^ and the reduction of MG in the presence of NADH, respectively. All strains grown in SD were harvested every 12 h to conduct biochemical experiments at both the midand the late-exponential growth phases. All experiments were conducted independently at least three times. Data are represented as the mean ± Sd of three independent experiments (*p < 0.05, **p < 0.01, ***p < 0.001).

To assess the effect of *MGD1* and *ADH1* expression in YB203 (*Δgcs1*) on antioxidative enzyme activities, the previously constructed *MGD1* and *ADH1* disruptants were used in this study [11, 12]; MK603 (*Δmgd1*/*Δgcs1*), MK703 (*Δadh1*/*Δgcs1*), and MK803 (*Δmgd1*/*Δadh1*/*Δgcs1*). In GSH-depleted conditions, all mutant strains showed negligibly low Mgd activities under all experimental conditions (Fig. 2A). However, compared to YB203 (*Δgcs1*), commonly decreased Mgr activity was shown in all disruptants; MK703 (*Δadh1*/*Δgcs1*) and MK803 (*Δmgd1*/*Δadh1*/*Δgcs1*) had a noticeably lower Mgr activity than YB203 (*Δgcs1*) (Fig. 2B). Thus, the Mgr activity of Adh1 was thought to be the main MG-scavenging enzyme in YB204 (*Δgcs1*)-derived disruptants, including MK603 (*Δmgd1*/*Δgcs1*, MK703 (*Δadh1*/*Δgcs1*), and MK803 (*Δmgd1*/*Δadh1*/*Δgcs1*). This result suggested that MG-reducing activity is the predominant MG detoxification strategy in *C. albicans*.

### Supraphysiological MG Accumulation in *Δmgd1*/*Δadh1*/*Δgcs1*

**Fig. 3:**
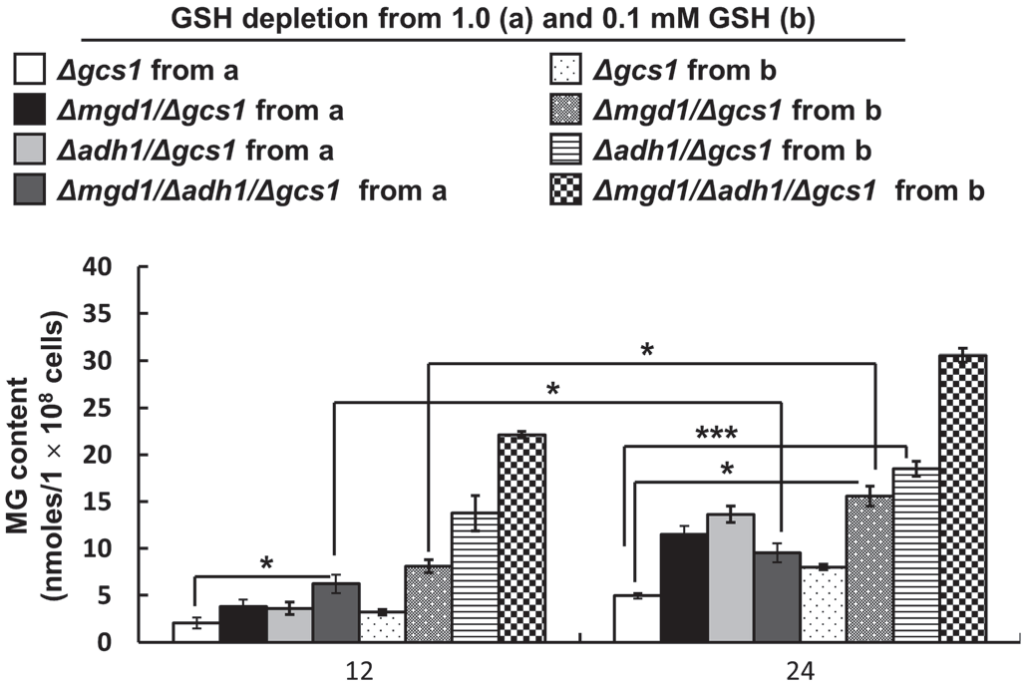
The intracellular contents of MG and pyruvate in *Δgcs1*-based disruptants. To monitor intracellular MG pyruvate contents, the *MGD1* and *ADH1* disruptants in *Δgcs1* were used to determine MG pyruvate content changes in SD liquid media. Exponentially growing cells at 18 h were transferred and regrown in minimally defined SD broth in this experiment. The values represent the averages ± standard deviation of three independent experiments.

Quinoxaline derivatives, 2-methylquinoxaline and 2-hydroxy-3-methylquinoxaline, were quantified in all disruptants (Fig. 3). As expected, the intracellular MG content significantly increased in all of the *Δgcs1*-based disruptants. However, while our previous observations suggested that Mgd1 and/or Adh1 activity deficiency accumulates MG and pyruvate without exception in MK603 (*Δmgd1*/*Δgcs1*) and MK703 (*Δadh1*/*Δgcs1*) [11, 12], a decrease in pyruvate was seen in MK803 (*Δmgd1*/*Δadh1*/*Δgcs1*). This result was not displayed in double gene-disrupted strains, including MK603 (*Δmgd1*/*Δgcs1*) and MK703 (*Δadh1*/*Δgcs1*). This metabolite content change behavior did not also coincide with previous experiments using the wild type-based disruptants (MK303 (*Δmgd1*) and MK403 (*Δadh1*), or Mgd1-overproducing mutants of MG and pyruvate in MK306 (*MGD1*^*OE*^) and MK606 ((*MGD1*^*OE*^)/*Δgcs1*) [11, 12]. The decrease in pyruvate content in MK803 (*Δmgd1*/*Δgcs1*) was assumed to be based on the absence of both *MGD1* and *ADH1*. This hypothesis was posited due to Mgd1 being responsible for catalyzing the reduction of pyruvate into lactate [12].

### Extremely Low Glr1 Activity with ROS Content in *Δgcs1*-Based Double and Triple Disruptants

**Fig. 4:**
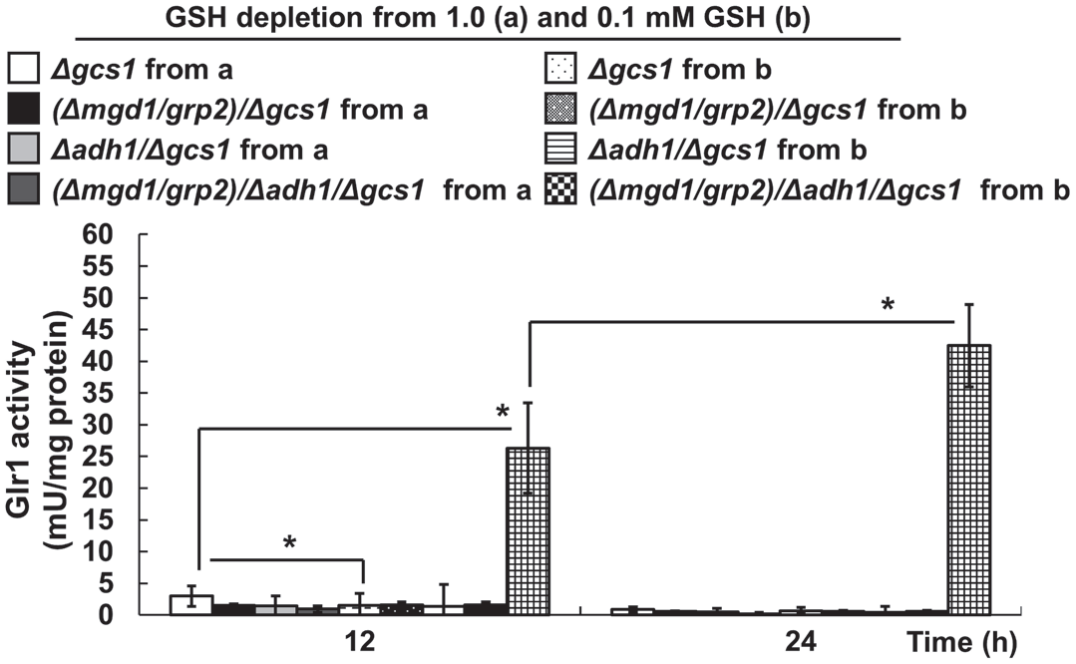
The effect of both *MGD1* and *ADH1* gene disruption on Glr1 activity in *MGD1* and/or *ADH1* disruptants. Cellular Glr1 activity was measured spectrophotometrically using continuous assays. One unit (U) of the enzyme is defined as described in the Materials and Methods section. Each experimental sample was compared to wild-type SC5314. As with the other experiments, *C. albicans* strains were commonly grown in SD liquid media. All experiments were conducted independently at least three times. Data are represented as the mean ± Sd of the experiments (*p < 0.05, **p < 0.01, ***p < 0.001).

Based on the extremely low GSH (Fig. 1) and supraphysiological MG levels in MK803 (*Δmgd1*/*Δgcs1*) (Fig. 3), we examined Glr1 activity’s association with Mgd1 and Adh1 activities (Fig. 4). All experimental groups were compared to GSH-depleted YB203 (*Δgcs1*)-derived disruptants. A negligibly low GSH content-derived Glr1 activity (Fig. 1) was seen in all disruptants. GSH-depleted MK603 (*Δmgd1*/*Δgcs1*) and MK703 (*Δadh1*) exhibited more severe defects in the Glr1 activity than YB203 (*Δgcs1*). The results strongly indicate that Glr1 activity is not involved the regulation of MK803 (*Δmgd1*/*Δgcs1*), and other YB203 (*Δgcs1*)-based disruptants, despite evident changes in MG and GSH seemingly being associated with GSH-dependent physiological states in our previous works [11, 12]. The data also suggest Glr1 activity- and GSH-independent redox states, particularly for MK803 (*Δmgd1*/*Δgcs1*).

**Fig. 5:**
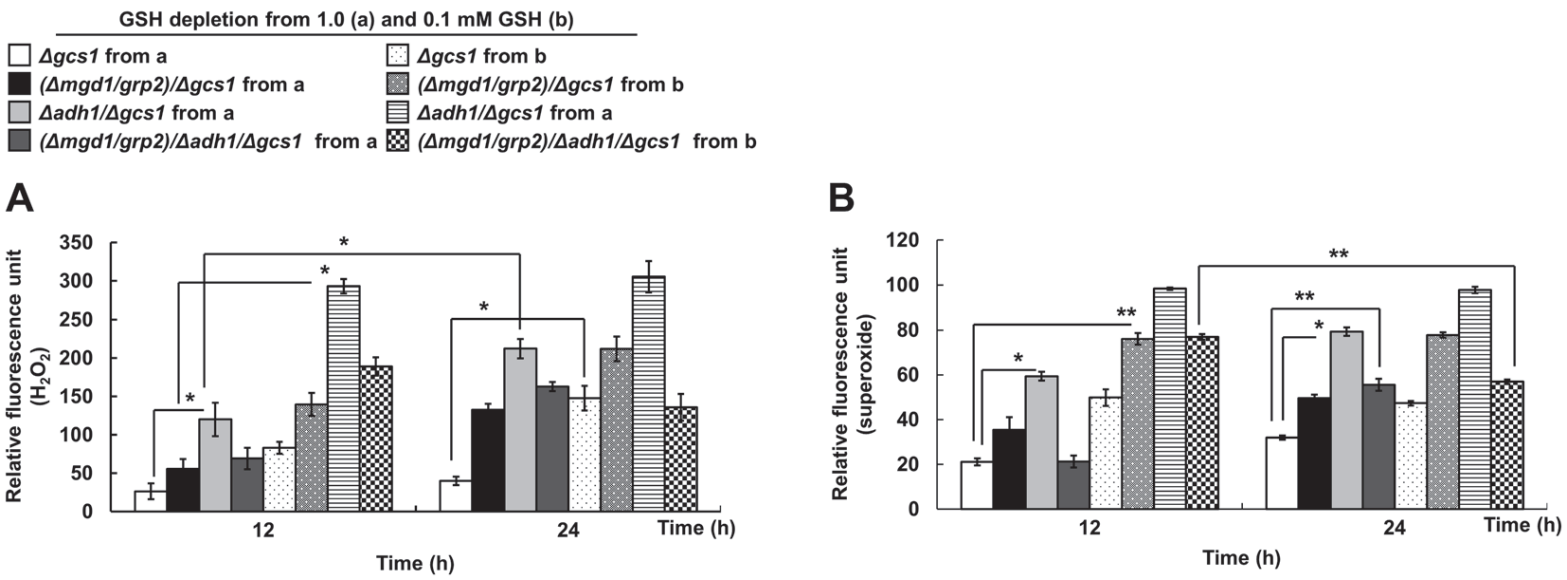
Cellular content of H_2_O_2_ and superoxide in *MGD1*- and/or *ADH1*-deficent Δgcs1-derived MK803 (Δmgd1/Δadh1/Δgcs1). The resulting cellular levels of (**A**) H_2_O_2_ and (**B**) Superoxide are suggested in these mutants. All *C. albicans* strains were grown in glucose-containing proliferation-inducing SD media. All budding strains in SD liquid media were cultured and harvested every 12 h to measure ROS content at both the mid- and the late-exponential growth phases. Exponentially grown cells at 18 h were transferred and regrown in minimally defined SD broth. All biological samples were prepared as described in Materials and Methods. Data are represented as the mean ± Sd of three independent experiments (*p < 0.05, **p < 0.01, ***p < 0.001).

To correlate between GSH-independent redox states and ROS accumulation in these mutants, cellular H_2_O_2_ (Fig. 5A) and superoxide (Fig. 5B) levels were determined using dihydroethidium and H_2_DCFDA, respectively. In the GSH- and Glr1 activity-independent redox states (Fig. 5), cellular ROS in MK603 (*Δmgd1*/*Δgcs1*) and MK703 (*Δadh1*/*Δgcs1*) significantly increased proportionally to MG accumulation (Fig. 3). However, cellular H_2_O_2_ and superoxide in MK803 (*Δmgd1*/*Δadh1*/*Δgcs1*) evenly decreased under all experimental conditions, especially in GSH-depleted MK803 (*Δmgd1*/*Δadh1*/*Δgcs1*) transferred from 0.1 mM GSH-containing SD liquid media in the late exponential phase (24 h). The data suggest, based on the absence of enzyme activities of both Mgd1 and Adh1, the existence of GSH-independent antioxidative defense mechanisms which are parallel to the MG-detoxifying functions.

### Antioxidative Eapx1 and Ccp1 Enzyme Activities in GSH-Depleted *Δgcs1*-Based Double and Triple Disruptants

**Fig. 6:**
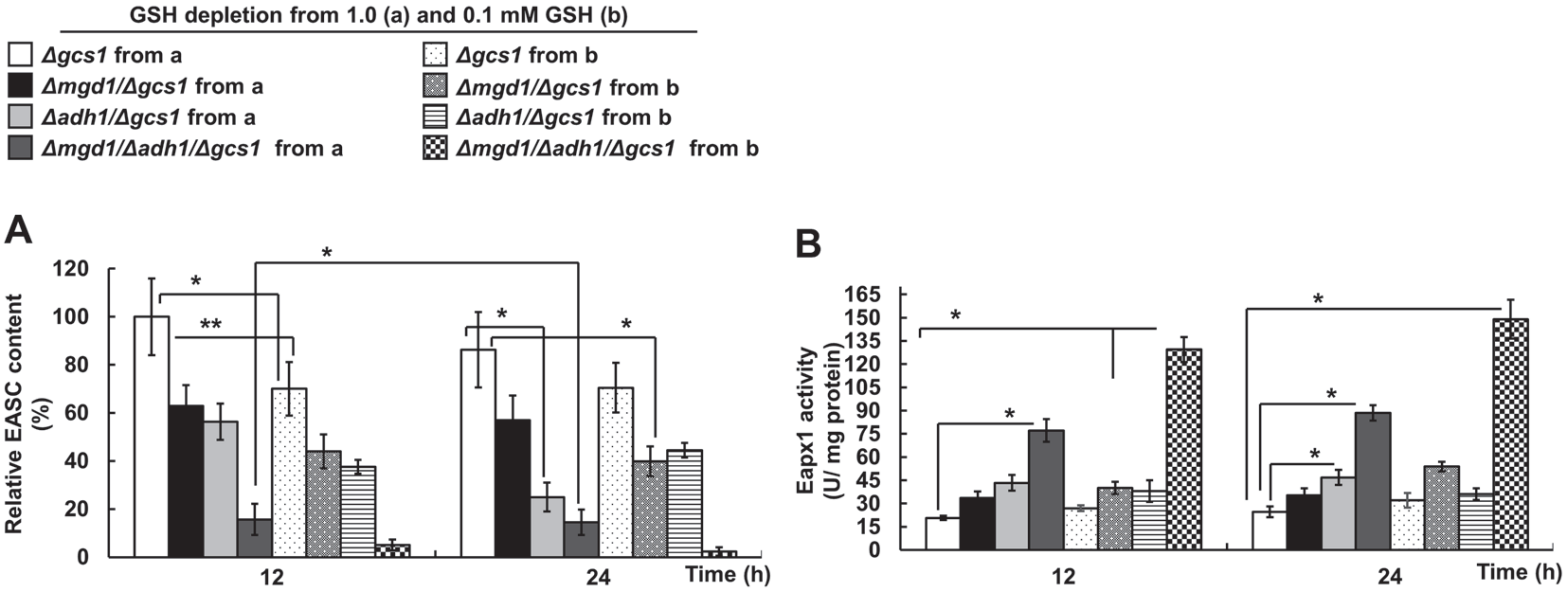
The effect of MK803 (*Δmgd1/Δadh1/Δgcs1*) on GSH-independent enzyme activities in *C. albicans*. The intracellular (**A**) EASC content and (**B**) its corresponding Eapx1 activity in *Δgcs1*-based disruptants. GSH-independent Eapx1 activity and EASC levels were compared during the mid and late exponentially growth phase of the cells in minimally defined SD broth. Data are represented as the mean ± Sd of three independent experiments (*p < 0.05, **p < 0.01, ***p < 0.001).

From the results of GSH- and Glr1 activity-independent redox regulation in MK803 (*Δmgd1*/*Δadh1*/*Δgcs1*), which revealed little Glr1 activity and cellular GSH levels with significant decreases in ROS (Figs. 3-5), we hypothesized the existence of an unbalanced redox pool. Cellular changes in ROS or MG production are thought to affect endogenous Eapx1 activity and its substrate D-erythroascorbic acid, which has been previously demonstrated in GSH-independent antioxidative materials [19]. In *Δgcs1*-based double and triple disruptants, independently of the cellular GSH level and Glr1 activity, MK603 (*Δmgd1*/*Δgcs1*) and MK703 (*Δadh1*/*Δgcs1*) transferred from 0.1 mM GSH-containing SD liquid media exhibited approximately 56.1 and 60.2% (12 h) and 62.5 and 55.5% (24 h) decreased D-erythroascorbic acid contents compared to *Δgcs1*, respectively (Fig. 6A). It was previously reported that calculated D-erythroascorbic acid values in these mutants coincided with concomitant changes in their cellular D-erythroascorbic acid and GSH content [19]. However, MK803 (*Δmgd1*/*Δadh1*/*Δgcs1*) transferred from 0.1 mM GSH-containing SD liquid media resulted in a decrease in D-erythroascorbic acid, mainly due to the absence of MG scavengers by gene disruption, falling to 95 and 97.7% at 12 and 24 h, respectively. In contrast with D-erythroascorbic acid content in all disruptants, Eapx1 activity saw a remarkable increase in all of the disruptants tested, including MK803 (*Δmgd1*/*Δadh1*/*Δgcs1*). Based on this finding, intracellular D-erythroascorbic acid in MK803 (*Δmgd1*/*Δadh1*/*Δgcs1*) seems to directly regulate ROS stress at a basal level, independently of the enzyme activities of Glr1, Mgd1, and Adh1. Our data are similar to those reported in our previous studies regarding alternative oxidase (*AOX*) gene expression along with D-arabinono-1,4-lactone oxidase (Alo1) activity [42], which also corresponded with the intracellular D-erythroascorbic acid level against the exogenous oxidative stress, or cyanide, components, participating in mitochondrial respiration in these mutants [42, 43].

**Fig. 7:**
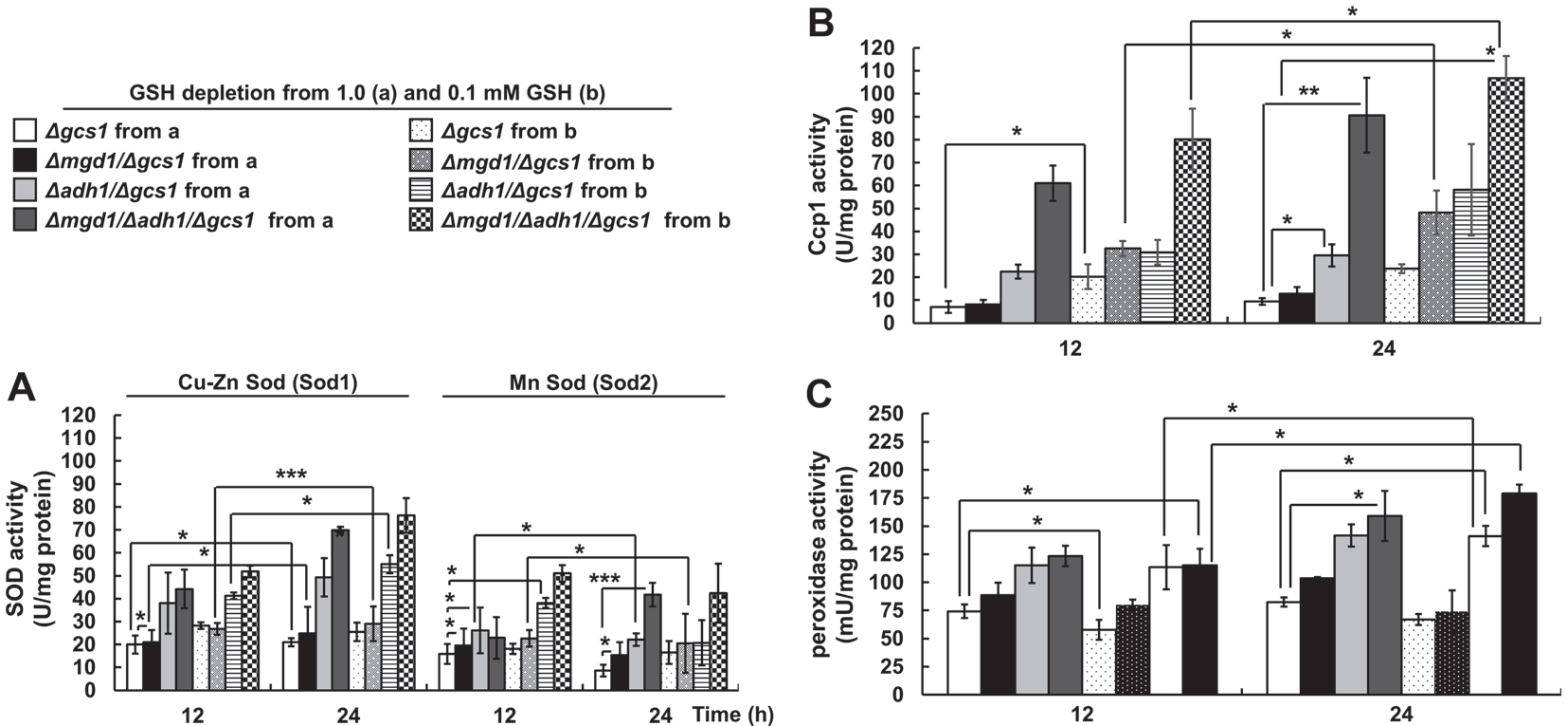
The effect of GSH-independent enzyme activities on Δgcs1-based disruptants in *C. albicans*. Enzyme activities of (**A**) Sod1 and Sod2, (**B**) Ccp1, and (**C**) total peroxidases in MK803 (*Δmgd1*/*Δadh1*/*Δgcs1*). Exponentially grown cells at 18 h were transferred and regrown in a minimally defined SD liquid medium at 28°C. All experiments were repeated at least three times. Error bars denote Sd. *p < 0.05, **p < 0.01, and ***p < 0.001, calculated using Student’s t-test.

Inspired by the results concerning the contribution of Eapx1 activity to cell viability (Fig. 6), we further examined the effects of the activities of other antioxidative defense enzymes, including Sod1, Sod2, Ccp1, and total peroxidases (Fig. 7). The enhanced antioxidative enzyme activities reflect poor redox states in all of the *MGD1* and *ADH1* disruptants. Particularly, Ccp1 activity in MK803 (*Δmgd1*/*Δadh1*/*Δgcs1*) was revealed to be the highest compared to that seen with other enzymes (*i.e.*, Sod1, Sod2, and total peroxidases) (Fig. 7B). Although the exact cellular mechanisms were elusive, the significantly elevated GSH-independent antioxidative activities against ROS increased, especially in the absence of MG scavengers. Our data corresponded with those of a previous investigation which saw that catalytic D-erythroascorbic acid oxidation in *ALO1*-deficient and *ALO1*-overexpressing cells maintained a parallel pathway only via *AOX* gene expression, irrelevant of endogenous H_2_O_2_ [42]. Nevertheless, cells lacking *APX* or *KatG* showed higher sensitivity against ROS, although metabolic genes such as pentose phosphate pathway genes, monodehydroascorbate reductase, and AOX in plants, were induced [44]. We concluded that the observed Eapx1 and Ccp1 enzyme activities, in combination with the basal utilization of cellular D-erythroascorbic acid, are the predominant GSH-independent ROS detoxification strategies in GSH-depleted *Δgcs1*-based double and triple disruptants. In addition, considering the accumulated MG in MK803 (*Δmgd1*/*Δadh1*/*Δgcs1*), GSH-independent ROS scavenging seems to be a parallel mechanism for MG-scavenging events. Our data also suggest that MG and ROS content can be affected by the pathophysiology- and virulence-controlling factors, Eapx1 and Ccp1, as reported [19, 20], use the reciprocal GSH-independent Eapx1 and Ccp1 activities in *C. albicans*. While *MGD1*-deficient cell physiology was regulated by *ADH1* expression, cellular MG content changes were convincingly controlled by both Mgd1 and Adh1 activities reciprocally during *Candida* cell growth.

## Discussion

The present study is based on the hypothesis that MG accumulation––through both the deprivation of MG-scavenging enzymes and induced GSH depletion––alters GSH-independent antioxidative enzyme activities. The study model, using Mgd1 and Adh1 in human pathogenic *C. albicans*, can provide an insight into another functional role for MG scavengers through the possible involvement of other physiological events altering the regulation of their interactive partners. Mgd1 consists of 341 amino acids with a molecular mass of 37,633.69 Da (http://web.expasy.org/compute_pi/). As one subunit of a monomer, this protein is a typical aldo–keto oxidoreductase [45], similar to Gre2 encoded by *S. cerevisiae* *YOL151w*. These enzymes are commonly induced by intracellular osmotic stress [46]. To avoid misleading information, because this protein has already been annotated as Grp2 based on a domain search of the *C. albicans* genome database, the previously characterized Mgd protein in our study has been designated as Mgd1 [12]. Interestingly, a conserved *Candida* cinnamyl Adh sequence and an NAD^+^-binding motif have been observed in Mgd1 and Adh1 (http://www.ncbi.nlm.nih.gov/Structure/cdd/wrpsb.cgi).

Thus, under the control of the absence of MG-consuming enzyme activities, we preferentially consider the stress–strain characteristics of *Δgcs1* in *C. albicans*. Fig. 1 shows non-viable *C. albicans* *Δgcs1* during GSH starvation. These data suggest a key role for GSH as a redox buffer in cells. To assess the effect of GSH depletion on cellular redox state via antioxidative enzyme activities in the absence of MG scavengers in this strain, nearly non-viable *Δgcs1* supplemented with 0.05 mM GSH was excluded from this study. This is due to there being extremely low cell numbers by cell lysis, especially in MK803 (*Δmgd1*/*Δadh1*/*Δgcs1*). Particularly, GSH-depleted *Δgcs1* transferred from 1 and 0.1 mM GSH-containing SD liquid medium was cultured for heterozygous Ura^+^ mutants for comparisons between antioxidative enzymes’ physiological and metabolic changes. However, we repeatedly failed to collect a sufficient number of cells from GSH-depleted triple disruptant MK803 (*Δmgd1*/*Δadh1*/*Δgcs1*) transferred from 0.1 mM GSH-containing medium (data not shown). The low cell numbers meant that bioassays for MG and pyruvate content could not be easily performed. Thus, Ura^+^ heterozygous MK803 (*Δmgd1*/*Δadh1*/*Δgcs1*) had to be grown in a 15-fold higher volume of SD broth than the other mutants. Importantly, in the case of GSH uptake below 1 mM GSH, supplementation in this study should be assumed to be three-fold greater than the GSH synthesis rate previously used for 1 mM amino acids against oxidative injury in isolated kidney cells [47]. This step was essential for observable GSH-depleted states in all disruptants with GSH-auxotrophic- and MG accumulation-characteristics, and because the intention was to convincingly provide data for GSH-independent antioxidative enzyme activities between MG-accumulating strains. When considering GSH physiology from *C. albicans* *Δgcs1*, GSH-auxotrophic characteristics have been noted in other eukaryotic microorganisms. These mutants essentially require exogenous GSH for cell proliferation [48, 49]. *C. albicans* *Δgcs1* displays complete GSH auxotrophy, with typical markers of apoptosis [21] and with stimulation of Adh1 activity [11]. Even in rich media, *Δgcs1*, with its GSH-auxotrophic nature, is seemingly more susceptible to certain types of α-ketoaldehydes (*i.e.*, glyoxal, MG, phenylglyoxal) and H_2_O_2_ than to reference strains [12]. These previous reports concur with our previous findings that demonstrated MG production by GSH deprivation and/or exogenous glucose treatment in the growth media resulted in growth defects with G1 phase-specific cell-cycle arrest in *D. dictyostelium* *alrA* and ornithine decarboxylase disruptants [50, 51]. Because the main experimental materials were constructed based on *Δgcs1* to accumulate cellular MG and pyruvate, this study excluded GSH-supplemented *Δgcs1* to emphasize the Mgd and Mgr activities in MK603 (*Δmgd1*/*Δgcs1*, MK703 (*Δadh1*/*Δgcs1*), and MK803 (*Δmgd1*/*Δadh1*/*Δgcs1*).

Our previous findings modeled in *Δgcs1* saw significantly enhanced Adh1 activity, catalyzing the oxidation of MG to pyruvate in GSH-depleted states [11]. Similarly, there is some evidence for the conversion of MG to pyruvate through NAD^+^-linked MG-oxidizing activities in *Δgcs1* [11, 12], which is contrary our hypothesis for predominant MG-reducing enzyme activities for Mgd1 and Adh1. However, this behavior was seen as similar to that of high Mgr activity in cell crude extracts, and was in sharp contrast with Mgd activity in *EAPX1* and *CCP1* double disruptants [20]. Moreover, the results seen in Fig. 2 are thought to be partially due to Mgd1 activity being responsible for the catalysis of the reduction of pyruvate into lactate [12]. Our data indicate that Mgd activity is much lower than natural Mgr properties in yeast. Data seen in Fig. 2 thus supports findings in previous reports that Mgd activity, in many gram-positive and gram-negative bacteria, does not seem to be required for cell physiology, while Mgr activity is [52]. Interestingly, Mgr activity in *S. cerevisiae* seems to have a similar profile as that seen in wild-type and GSH-supplemented *Δgcs1* in *C. albicans* [52].

Fig. 3 illustrates a remarkable decrease in the pyruvate content of MK803 (*Δmgd1*/*Δgcs1*) when compared to pyruvate accumulation in MK603 (*Δmgd1*/*Δgcs1*) and MK703 (*Δadh1*/*Δgcs1*). MK603 (*Δmgd1*/*Δgcs1*) and MK703 (*Δadh1*/*Δgcs1*) commonly experienced cellular accumulation of MG and pyruvate. This result is in agreement with our previous works indicating that increases in pyruvate in YM203 (*Δgcs1*) and its-derived MG scavengers’ disruptants results from GSH depletion in *Δgcs1*. These data also correspond with a previous finding that the human neuroblastoma cell line SH-SY5Y, treated with a non-lethal dose of exogenous MG, accumulates both lactate and pyruvate owing to a limitation of intracellular utilization via the TCA cycle [53]. However, the previous and present results concerning pyruvate accumulation, do not fully correspond with our previous works, because pH-dependent Mgd1 activity favors NADH oxidation at near-physiological pH, ranging from 5.5 to 7.5, as against NAD^+^ reduction at a pH of greater than 8 [12]. In addition, the Mgd1 activity at a near-neutral pH in its overproducing strains, including in MK306 (*MGD1*^*OE*^) and MK606 ((*MGD1*^*OE*^)/*Δgcs1*), also appears to preferentially catalyze MG reduction to acetol rather than oxidation to pyruvate [12], because *C. albicans* cells normally grow in acidic environments and laboratory growth media, such as seen in the minimally defined SD used in the current study, having a pH range of 5.5–6 [54]. Thus, in the case of the reduction activity of Mgd1 and Adh1 in nature, the increase in the enzyme activities of these MG scavengers seems to be accompanied by a decrease in pH during growth. Meanwhile, an increase in pH of the culture medium is required for NAD^+^-linked Mgd1 and Adh1 activity. Nevertheless, as a piece of convincing evidence of the predominant reduction activity of Mgd1 and Adh1, a previous study suggested loss of GSH leading to an intracellular pH decrease was due to a reversible impairment of the Na^+^/H^+^ antiporter; the main system for the maintenance of pH during the replication of the Sendai virus in Madin–Darby canine kidney cells [55]. Importantly, when looking at MG reduction activity in crude extracts (Fig. 2), the inducible Mgd1 and Adh1 activities seem to switch their catalytic activities as driven by changes in cellular GSH, MG, NAD^+^/NADH ratios resulting from the natural characteristics of our mutants. Also, other possible relevant metabolites, presumably dependent on the redox state and intra- and extracellular pH changes during cell growth, are hypothesized to be involved in MG metabolism. Thus, the decrease in pyruvate in MK803 (*Δmgd1*/*Δgcs1*) is thought to predominantly come from the absence of both genes *MGD1* and *ADH1*.

The negligibly low GSH content (Fig. 1), and thus Glr1 activity (Fig. 4), mean GSH-independent redox control by the disruptants lacking *MGD1* and *ADH1*. This result can be supported by observable decreases in GSH and Glr1 activity in monoterpene phenol-treated *C. albicans* [56]. Considering the low GSH uptake rate in *Δgcs1* (Fig. 1), we expected GLR1 expression enhancement to complement cellular GSH in the *Δgcs1*-based disruptants in this study. However, our data regarding cellular GSH content and Glr1 activity in all disruptants (Figs. 1 and 4) contrast with notable increases seen in Glr1 activity-triggered oxidative-stress defense signaling in cytochrome c peroxidase (*CCP*) mutants in *S. cerevisiae*, including *ccp1Δ* and *ccp1Δ*-ccp1W191F, with increased H_2_O_2_ resistance [57].

We have previously reported on ROS-triggered antioxidative defense enzymes, including Sod1, Sod2, *KatG*, Eapx1, and Ccp1 [20, 58], along with antioxidative activities of Mgd1 and Adh1 [11, 12]. These antioxidative enzymes, except for the manganese-containing Sod2 disruptant *sod2*/*sod2* which does not show virulent characteristics, are commonly related to virulence and hyphal formation in *C. albicans* [59]. This latter report suggests that the pathophysiological function of Sod1 differs from that of Sod2 in *C. albicans*. However, only some antioxidative mechanisms for Sod1 and Sod2 have been reported on the pathogenicity of the *C. albicans* wild type strain SC5314 in budding and filamentous growth, not with GSH-independent *Δgcs1*. As a representative example, cellular D-erythroascorbic acid generally activates cyanide-resistant respiration by the induction of alternative oxidase (*AOX*) gene expression in wild-type cells. However, the *AOX* gene is not known to be responsive to D-erythroascorbic acid-biosynthesizing gene expression independently of arabinono-1,4-lactone oxidase (Alo1) [42]. Thus, D-erythroascorbic acid-mediated *AOX* gene expression is independent of cellular H_2_O_2_ content in budding and hyphal-growing *C. albicans*. Also, D-erythroascorbic acid oxidation is less sensitive against exogenous H_2_O_2_ when compared to other oxidants. Moreover, cellular D-erythroascorbic acid content does not always affect cellular GSH content in *S. cerevisiae* [60]. These results suggest that ascorbic acid or D-erythroascorbic acid has a limited protective role against oxidative stress in yeast cells [42, 60]. Meanwhile, *Δgcs1*-driven disruptants [*i.e.*, MK603 (*Δmgd1*/*Δgcs1*), MK703 (*Δadh1*/*Δgcs1*), and MK803 (*Δmgd1*/*Δadh1*/*Δgcs1*)] undergo severe growth defects with supraphysiological accumulation of both MG and ROS (Figs. 3-5). The behavior of these mutants illustrates another crucial role for GSH- and Glr1 activity-independent ROS detoxification by Eapx1 and Ccp1 among antioxidative enzymes, parallel to activities of MG scavengers. GSH-independent antioxidative activities from our mutants with decreased cellular ROS in *Δgcs1*-based mutant MK803 (*Δmgd1*/*Δadh1*/*Δgcs1*) can be also sufficiently supported by previous investigations in plants where chloroplastic peroxidases- and/or *APX*-deficient plants commonly lost their ASC-oxidizing activity, which predominantly regulates cellular H_2_O_2_ [61]. Meanwhile, they display severe cellular damage by H_2_O_2_ [62]. Also, a chloroplastic homologue of mitochondrial *AOX* gene expression is known to be significantly induced in *APX*-deficient cells [44]. In our previous study, elevated endogenous ROS reciprocally increased cellular MG content [11]. These reports illustrate that cellular GSH content does not contribute to cellular stress resistance, because cellular ROS stress is thought to be readily and drastically altered by other redox buffering mechanisms in fungi [63]. Importantly, in vitro spectrophotometric observations suggest hydrogen bonded complex formation between MG and ASC [64]. Thus, this study demonstrates the predominant antioxidative properties of Eapx1 and Ccp1 through *Δgcs1*-based mutant MK803 (*Δmgd1*/*Δadh1*/*Δgcs1*), in the absence of both GSH redox regulation and Mgd1 and Adh1 activities. Therefore, D-erythroascorbic acid-oxidizing Eapx1, Ccp1, or other peroxidases may be engaged in redox homeostasis, independently of GSH-linked redox regulation, via the enzymatic activities of MG scavengers during GSH depletion.

Further studies are needed to consider whether the parallel mechanism seen with MK803 (*Δmgd1*/*Δadh1*/*Δgcs1*) is similar to that of Adh1 in our previously demonstrated data [12], and whether, independently of GSH and Glr1 activities in *C. albicans*, this accompanies the glycolytic enzymes Fba1 and Tdh3. Also, GSH-depleted MK803 (*Δmgd1*/*Δadh1*/*Δgcs1*) might maximize cellular MG accumulation, affecting glycolysis and mitochondrial respiration, through the loss of the multifunctional activity of MG detoxification. In addition, based on our previous work concerning Mgd-like proteins, including aldolase and hexokinase in *MGD1*-deficient GSH-depleted cells as compared to wild-type and GSH-supplemented strains in terms of MG detoxification, cellular MG in GSH-depleted MK803 (*Δmgd1*/*Δadh1*/*Δgcs1*) might well be scavenged by other types of ADH-like proteins and glycolytic enzymes.

Taken together, *MGD1* and *ADH1* deficiency of MK803 (*Δmgd1*/*Δadh1*/*Δgcs1*) regulate key antioxidative enzymes, independently of GSH biosynthesis with the aid of *ADH1* expression in *C. albicans*. Until now, there has been no experimental evidence for GSH-independent redox regulation using MG oxidoreductase mutants in GSH-depleted cells. This is a novel finding with little direct literature support. An *MGD1*-deficient GSH-depleted *Δmgd1*/*adh1*/*Δgcs1* strain has been examined, allowing for the estimation of cellular MG and pyruvate changes associated with poor GSH-independent redox states and possible glycolytic or mitochondrial changes. This strain has provided, and continues to provide, an excellent experimental model for creating environmental conditions specific for the induction of endogenous MG or pyruvate production during glycolysis in in vitro MG synthesis studies using GSH-deprived GSH auxotrophic mutants [65, 66].

## Conclusions

This study confirms that the GSH-depleted MK803 (*Δmgd1*/*Δadh1*/*Δgcs1*) undergoes defects in cell growth, an increase in MG production, and a decrease in cellular pyruvate, D-erythroascorbic acid, and ROS, which is in contrast to other *Δgcs1*-based *MGD1*- and *ADH1*-double gene-disrupted mutants. This model shows that the *MGD1*- and *ADH1*-deficient triple gene-disrupted strains evidently undergo metabolic changes in cellular MG. These results contrast with those of our previous studies on the *MGD1* single gene-disrupted strain and its overexpressing mutants, in the absence of Mgd and Glr1 activities. Of note, the inactivation of the activities of both Mgd1 and Adh1 triggers GSH-independent antioxidative enzymes, such as Eapx1 and Ccp1, in non-hyphae-inducing cells. This behavior of GSH-depleted MK803 (*Δmgd1*/*Δadh1*/*Δgcs1*) prominently suggests another crucial role for GSH- and Glr1 activity-independent ROS detoxification by antioxidative enzymes, especially for Eapx1 and Ccp1 in parallel with the activities of MG scavengers. Our findings also hint at the possibility that GSH-independent defense mechanisms against oxidative stress exist in the absence of MG scavengers.

## Figures and Tables

**Table 1:** Strains, primers, and plasmids used in this study.

Strain, primer, or plasmid	Genotype, sequence, or description	Source or reference
*C. albicans*		
SC5314	Wild type isolate	Fonzi and Irwin, 1993
CAI4	*Δura3::imm434/Δura3::imm434*	Fonzi and Irwin, 1993
YB203, heterozygous *Δgcs1*	*Δura3::imm434/Δura3::imm434 Δgcs1::hisG/Δgcs1::hph-URA3-hph*	Baek *et al.*, 2004
YB204, homozygous *Δgcs1*	*Δura3::imm434/Δura3::imm434 Δgcs1::hisG/Δgcs1::hph*	Baek *et al.*, 2004
MK601, heterozygous (*MGD1*/*mgd1*)/*Δgcs1*	As YB204, but *Δura3::imm434/Δura3::imm434 Δmgd1::hph-URA3-hph/MGD1*	Kwak *et al*., 2018
MK602, homozygous (*MGD1*/*mgd1*)/*Δgcs1*	As YB204, but *Δura3::imm434/Δura3::imm434 Δmgd1::hph/MGD1*	Kwak *et al*., 2018
MK603, heterozygous *Δmgd1*/*Δgcs1*	As YB204, but *Δura3::imm434/Δura3::imm434 Δmgd1::hph/Δmgd1::hph-URA3-hph*	Kwak *et al*., 2018
MK604, homozygous *Δmgd1*/*Δgcs1*	As YB204, but *Δura3::imm434/Δura3::imm434 Δmgd1::hph/Δmgd1::hph*	Kwak *et al*., 2018
MK701, heterozygous (*ADH1*/*adh1*)/*Δgcs1*	As YB204, but *Δadh1::hph-URA3-hph*/*ADH1*	Kwak *et al*., 2014
MK702, homozygous (*ADH1*/*adh1*)/*Δgcs1*	As YB204, but *Δadh1::hph*/*ADH1*	Kwak *et al*., 2014
MK703, heterozygous *Δadh1*/*Δgcs1*	As YB204, but *Δadh1::hph/Δadh1::hph-URA3-hph*	Kwak *et al*., 2014
MK704, homozygous *Δadh1*/*Δgcs1*	As YB204, but *Δadh1::hph/Δadh1::hph*	Kwak *et al*., 2014
MK801, heterozygous *Δmgd1*/(*ADH1*/*adh1*)/*Δgcs1*	As MK604, but *Δura3::imm434/Δura3::imm434 Δadh1::hph-URA3-hph/ADH1*	Kwak *et al*., 2018
MK802, homozygous *Δmgd1*/(*ADH1*/*adh1*)/*Δgcs1*	As MK604, but *Δura3::imm434/Δura3::imm434 Δadh1::hph/ADH1*	Kwak *et al*., 2018
MK803, heterozygous *Δmgd1*/*Δadh1*/*Δgcs1*	As MK604, but *Δura3::imm434/Δura3::imm434 Δadh1::hph/Δadh1::hph-URA3-hph*	Kwak *et al*., 2018
MK804, homozygous *Δmgd1*/*Δadh1*/*Δgcs1*	As MK604, but *Δura3::imm434/Δura3::imm434 Δadh1::hph/Δadh1::hph*	Kwak *et al*., 2018
Primers		
MK1a-SacI	5′-GAGCTCGGATAACTCTTCGTTTTATCCGTC-3′, SacI site of pMK1F	Kwak *et al*., 2018
MK1b-KpnI	5′-GGTACCATATGAAGATATTTTTTAATTGAT-3′, KpnI site of pMK1F	Kwak *et al*., 2018
MK1c-SalI	5′-GTCGACATGATATTCAGATTGAATGATGAT-3′, SalI site of pMK1R	Kwak *et al*., 2018
MK1d-HindIII	5′-AAGCTTATATTTGATCGCGAAGCAGATGTC-3′, HindIII site of pMK1R	Kwak *et al*., 2018
MK1e-BglII	5′-AGATCTATGTCTTCATCTACTACAGTTTTC-3′, BglII site of pMK1E	Kwak *et al*., 2018
MK1f-XhoI	5′-CTCGAGTTAACCAATAATTTGAGCAACCGA-3′, XhoI site of pMK1E	Kwak *et al*., 2018
MK2a-SacI	5′-GAGCTCTTACAATATTTGATAGAGACCCAA-3′, SacI site of pMK2F	Kwak *et al*., 2014
MK2b-KpnI	5′-GGTACCAAGTGCGGGATTATCCTTTTTGAG-3′, KpnI site of pMK2F	Kwak *et al*., 2014
MK2c-SalI	5′-GTCGACAAATAGCTAAATTATATACGAATT-3′, SalI site of pMK2R	Kwak *et al*., 2014
MK2d-HindIII	5′-AAGCTTAAACTTGAAAACACCGAGTTGATA-3′, HindIII site of pMK2R	Kwak *et al*., 2014
Plasmids		
pQF181	pUC18 containing *hph-URA3-hph* (forward) from pQF86	Kwak *et al*., 2018
pQF182	pUC18 containing *hph-URA3-hph* (reverse) from pQF86	Kwak *et al*., 2018
pMK1F	pGEM-T Easy vector containing MK1a-MK1b fragment, upstream region of *MGD1*	Kwak *et al*., 2018
pMK1R	pGEM-T Easy vector containing MK1c-MK1d fragment, downstream region of *MGD1*	Kwak *et al*., 2018
pMK2F	*MGD1* deletion construct with *hph-URA3-hph* (forward)	Kwak *et al*., 2018
pMK2R	*MGD1* deletion construct with *hph-URA3-hph* (reverse)	Kwak *et al*., 2018
pMK3F	pGEM-T Easy vector containing MK2a-MK2b, upstream region of *ADH1*	Kwak *et al*., 2014
pMK3R	pGEM-T Easy vector containing MK2c-MK2d, downstream region of *ADH1*	Kwak *et al*., 2014
pMK4F	*ADH1* deletion construct with *hph-URA3-hph* (forward)	Kwak *et al*., 2014
pMK4R	*ADH1* deletion construct with *hph-URA3-hph* (reverse)	Kwak *et al*., 2014
